# Supplementary material for: Ophiopogon Polysaccharide Promotes the In Vitro Metabolism of Ophiopogonins by Human Gut Microbiota
Source: Molecules. 2019 Aug 8;24(16):2886. doi: 10.3390/molecules24162886 (PMC6719028; doi:10.3390/molecules24162886)
Supplement: Supplementary file 1 [file molecules-24-02886-s001.pdf]

Supplementary Materials

Figure S1. *Ophiopogon* polysaccharide promotes the metabolism of ophiopogonin D and ophiopogonin D′, and the production of ruscogenin and diosgenin. The $AUC_{0\text{-}72h}$ of ophiopogonin D, ruscogenin, ophiopogonin D′ and diosgenin in different groups. Normal: ophiopogonin group; OJP: *Ophiopogon* polysaccharide (OJP) + ophiopogonin group. The area under the concentration–time curve ($AUC_{0\text{-}72h}$) were analyzed by Graphpad Prism software (v7.0). Data are represented as mean ± SD (n = 5). Significance difference was assessed by one-way ANOVA: $^{*}P < 0.05$ and $^{**}P < 0.01$ vs normal group.

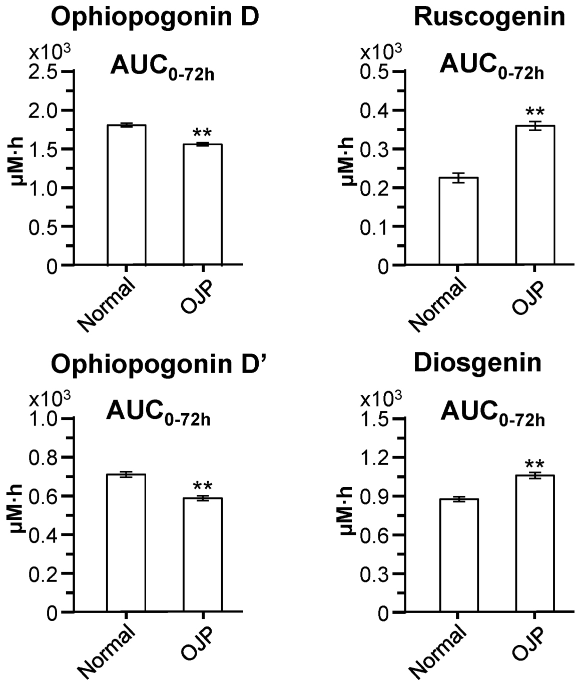

Table S1. Concentrations of ophiopogonin D, ophiopogonin D′ and their metabolites (i.e. ruscogenin and Diosgenin) in ten time points of normal group and OJP group.

Analyte	Group	Concentrations of ophiopogonins (μM): mean ± SD (*n* = 5) [c]									
		**0 h**	**6 h**	**12 h**	**18 h**	**24 h**	**30 h**	**36 h**	**48 h**	**60 h**	**72 h**
Ophiopogonin D	OJP [a]	30.53 ± 0.54	30.08 ± 0.84	29.04 ± 0.65	27.36 ± 1.14	23.31 ± 0.79	22.72 ± 1.10	21.72 ± 0.88	20.01 ± 1.31	17.49 ± 1.19	10.86 ± 1.17
	Normal [b]	30.61 ± 0.65	30.07 ± 1.08	29.67 ± 0.89	28.09 ± 1.33	27.43 ± 1.35	25.78 ± 1.03	25.13 ± 1.38	24.19 ± 1.56	23.36 ± 1.58	18.36 ± 1.36
Ruscogenin	OJP	ND [d]	ND	ND	0.60 ± 0.03	2.00 ± 0.20	4.12 ± 0.50	4.93 ± 0.46	6.87 ± 0.52	8.94 ± 0.90	14.53 ± 0.92
	Normal	ND	ND	ND	ND	0.90 ± 0.09	2.25 ± 0.34	3.53 ± 0.54	4.81 ± 0.83	5.62 ± 0.77	8.48 ± 1.15
Ophiopogonin D′	OJP	30.01 ± 0.77	26.95 ± 1.54	20.24 ± 1.34	17.45 ± 1.69	11.97 ± 1.19	1.63 ± 0.17	0.84 ± 0.01	0.80 ± 0.08	0.72 ± 0.06	0.63 ± 0.02
	Normal	29.53 ± 0.95	27.48 ± 1.37	25.93 ± 1.54	24.01 ± 1.90	17.39 ± 1.59	2.30 ± 0.26	1.55 ± 0.27	1.02 ± 0.07	0.85 ± 0.10	0.78 ± 0.04
Diosgenin	OJP	ND	ND	0.41 ± 0.03	1.51 ± 0.15	9.91 ± 0.86	18.42 ± 1.02	20.28 ± 1.18	21.51 ± 1.11	22.76 ± 1.68	28.72 ± 2.10
	Normal	ND	ND	ND	0.17 ± 0.04	8.45 ± 0.11	16.51 ± 0.67	17.60 ± 0.93	18.64 ± 1.25	18.90 ± 1.05	20.76 ± 1.63

[a] OJP: *Ophiopogon* polysaccharide (OJP) + ophiopogonin group; [b] Normal: ophiopogonin group; [c] Data are represented as mean ± SD (*n* = 5).; [d] ND: not detected.
